# Procoagulant Activity of Blood and Microvesicles Is Disturbed by Pneumococcal Pneumolysin, Which Interacts with Coagulation Factors

**DOI:** 10.1159/000525479

**Published:** 2022-07-15

**Authors:** Sonja Oehmcke-Hecht, Claudia Maletzki, Surabhi Surabhi, Nikolai Siemens, Valeria Khaimov, Lisa Marie John, Sina Mariella Peter, Sven Hammerschmidt, Bernd Kreikemeyer

**Affiliations:** ^a^Institute of Medical Microbiology, Virology and Hygiene, Rostock University Medical Center, Rostock, Germany; ^b^Department of Medicine, Clinic III-Hematology, Oncology, Palliative Medicine, Rostock University Medical Center, Rostock, Germany; ^c^Department of Molecular Genetics and Infection Biology, Interfaculty Institute for Genetics and Functional Genomics, Center for Functional Genomics of Microbes, University of Greifswald, Greifswald, Germany; ^d^Institute for ImplantTechnology and Biomaterials e.V., Rostock, Germany

**Keywords:** Pneumolysin, Coagulation, Contact system, Microvesicles

## Abstract

The coagulation and contact systems are parts of the innate immune system as they prevent bleeding and dissemination of pathogens and also contribute to microbial killing by inflammatory reactions and the release of antimicrobial peptides. Here, we investigated the influence of *Streptococcus pneumoniae* on the coagulation and contact system. *S. pneumoniae* (pneumococci), but no other investigated streptococcal species, impairs coagulation of blood by autolysis and release of pneumolysin. Defective blood coagulation results from the lysis of tissue factor-producing mononuclear cells and their procoagulant microvesicles, which are the main trigger for blood coagulation during sepsis. In addition, pneumolysin binds coagulation and contact system factors, but this does not result in activation. Thus, pneumococci modulate activation of the coagulation system by releasing pneumolysin, which could potentiate lung injury during pneumonia.

## Introduction

*Streptococcus pneumoniae* (the “pneumococcus”) is a major human pathogen responsible for a range of severe diseases such as pneumonia, meningitis, and bacteremia. Pneumococci produce pneumolysin, a toxin, which is expressed by nearly all clinical isolates and has been described as the key virulence factor contributing to high morbidity and mortality rates in invasive disease [1]. Pneumolysin is a cholesterol-dependent cytolysin, which lyses host cells with cholesterol in their membranes, activates host complement, and induces proinflammatory reactions in immune cells [2, 3, 4]. Pneumolysin causes widespread cellular and tissue damage, triggering increased bacterial replication and tissue invasion. The release of pneumolysin from the intracellular bacterial compartment occurs mainly via autolysis of pneumococci. Autolysis is induced by the major autolysin LytA, which is a peptidoglycan hydrolase [5]. Mutants deficient in pneumolysin or LytA are attenuated in virulence after intranasal administration into mice [6, 7]. Thus, pneumolysin and autolysin are important virulence determinants of pneumococci, and increased pathogenicity of some pneumococcal strains is driven by their properties of rapid autolysis and the massive release of pneumolysin [8].

The presence of pneumolysin in the lung facilitates penetration of bacteria from the alveoli into the interstitium of the lung and dissemination of pneumococci into the bloodstream [9]. Moreover, the cytotoxic effect of pneumolysin on the alveolar epithelium induces acute lung injury, with massive alveolar hemorrhage [10]. It is the hemorrhage, not inflammation, which led to potentiation of lung injury and increased mortality [11]. The mechanisms that contribute to alveolar hemorrhage are not yet fully understood; however, two studies strongly suggest that impaired hemostasis is responsible [11, 12]. Hemostasis is a defense mechanism; it prevents bleeding and blood loss after injury. In addition, it contributes to innate immunity by trapping invading bacteria at the side of infection [13]. Hemostasis is a two-step process. First, platelets seal occurring gaps in the endothelium [14], and in the second step, the coagulation cascade becomes activated. Recently, Jahn et al. [12] showed that pneumolysin induces pores in platelets, which renders platelets nonfunctional and impairs formation of a thrombus and sealing of the endothelium. Coagulation activation after thrombus formation however ends in the generation of a fibrin network that functions as glue for the growing thrombus. Coagulation is primarily driven by tissue factor and the extrinsic coagulation pathway [15]. Intriguingly, if tissue factor-dependent activation of coagulation is reduced, alveolar hemorrhage and death in influenza A virus-infected mice occur [16]. Moreover, low levels of tissue factor also led to alveolar hemorrhage, potentiated murine acute lung injury and oxidative stress after intratracheal application of LPS [17]. This indicates that a functioning coagulation is essential to prevent bleeding and lung damage in infection and inflammatory conditions.

While the extrinsic coagulation pathway is essential for physiological coagulation, this is not the case with the intrinsic pathway (also called the contact system). However, binding of the three contact factors high-molecular-weight kininogen (HK), factor XII (FXII), and plasmakallikrein (PK) at bacterial surfaces can activate the system. Activation results in inflammatory reactions and the release of antimicrobial peptides and therefore contributes to innate immunity (for a review see [18]).

Thus, impaired coagulation and contact system activation, especially in the beginning of the disease, might support lung hemorrhage and damage. The objective of this study was to investigate how pneumococci interact and modulate the coagulation and contact system. We show that exclusively pneumococci, but no other investigated streptococcal species, impair coagulation of blood by autolysis and release of pneumolysin. Defective blood coagulation results from the lysis of blood cells and their procoagulant microvesicles (MVs), which are the main triggers for blood coagulation during sepsis [19]. Beside this, pneumolysin binds the contact system proteins FXII, PK, and HK, but this does not activate the contact system. Thus, pneumococci impair activation of the coagulation system mainly by the release of pneumolysin, which might worsen severe lung hemorrhage.

## Materials and Methods

### Bacterial Strains and Culture Conditions

Bacterial strains are listed in Table 1. Bacteria were grown on blood agar plates at 37°C, aerobically, overnight and subsequently stored at 4°C. For further use, overnight cultures of *S. pyogenes, S. gallolyticus*, and *S. suis* were cultivated in in Todd-Hewitt broth supplemented with 0.5% yeast extract (Roth) at 37°C under a 5% CO_2,_ 20% O_2_ atmosphere, centrifuged (2,500 *g*, 5 min), washed twice in phosphate buffered saline (PBS) and set to the desired CFU/mL. *S. pneumoniae* D39 and TIGR4 and their isogenic mutants were grown on blood agar plates and cultured in Todd-Hewitt broth, supplemented with 0.5% yeast extract containing erythromycin (5 μg mL^−1^) and/or chloramphenicol (8 μg mL^−1^) if required. Cultivation of pneumococci on blood agar or in liquid cultures was performed at 37°C and 5% CO_2_ without agitation and for a maximum of 9 h, centrifuged (2,500 *g*, 5 min), washed twice in PBS or HEPES, and set to the desired CFU/mL.

### Pneumolysin Production and Cytolytic Activity

Purification of recombinant pneumolysin has been done as described before [12, 24] and were shown to be endotoxin-free by this method. Cytolytic activity of purified pneumolysin was tested by hemolysis of erythrocytes from healthy human volunteers. Blood was incubated with pneumolysin or bacterial supernatants for 60 min at 37°C in a 96-well plate (U-bottom). After incubation, the plate was centrifuged and monitored for formation of the erythrocyte sediment. Alternatively, the absorbance of the supernatant at 540 nM was quantified after centrifugation.

### Quantification of Pneumolysin in Pneumococci Culture Supernatants

*S. pneumoniae TIGR4* and *TIGR4∆ply* were grown as described above, washed twice in HEPES, and set to 1 × 10^10^ CFU/mL 480 µL bacteria was incubated in 480 μL HEPES for 4 h at 37°C. Supernatants were collected for immunoblotting. In addition, a serial dilution of recombinant pneumolysin protein was used as standard. The samples were blotted on a nitrocellulose membrane, and after blocking, the anti-pneumolysin antibody (Davids Biotechnologie GmbH) was incubated. After washing, blots were incubated with secondary ﬂuorophore-labeled antibodies (LI-COR) and imaged on Odyssey Imager (LI-COR). Pneumolysin relative protein levels in the supernatant were determined by densitometry analysis (EmpiraStudio, LI-COR). Calculation of the pneumolysin amount based on the pneumolysin standard curve was performed with Microsoft Excel (Version16.6.).

### Human Plasma

Pooled plasma obtained from 20 healthy donors was purchased from Affinity Biologicals Inc. (Canada).

### Preparation and Treatment of PBMCs

PBMCs were isolated using diluted blood (1:1 in PBS) from healthy volunteers as described [27]. The PBMC cell layer was collected, and cells were washed twice in PBS and resuspended in RPMI medium (Invitrogen). PBMCs (1 × 10^6^ cells/mL) were treated with pneumolysin or autolysin at indicated concentrations or LPS (Sigma-Aldrich) at 1 μg/mL. Medium alone was used as the control. After an overnight incubation (20 h) on rotation at 37°C, cells were centrifuged (400 *g* for 20 min), resuspended in 100 μL PBS, and used for clotting assays. Supernatants were kept frozen (−80°C) until use.

### Isolation of MVs from PBMCs

Stimulation of PBMCs with LPS and isolation of procoagulant MVs were done as described before [28, 29].

### Cytokine ELISA

The concentrations of IL-6, TNF-alpha, and IL10 in the supernatants from PBMCs were determined by ELISA according to the manufacturer's protocol (R&D Systems).

### Blood and PBMC Phenotyping

Blood or PBMCs (1 × 10^6^ cells) were taken from healthy volunteers and incubated with indicated pneumolysin concentration for 4 h (blood) or 20 h (PBMCs) at 37°C. Thereafter, single cells were stained with a panel of conjugated monoclonal antibodies (1.0 μg each, 30 min, 4°C) followed by lysis (155 mM NH_4_Cl [MERCK Millipore, Darmstadt, Germany]), 10 mM KHCO3 (MERCK Millipore), and 0.1 mM EDTA (AppliChem, Darmstadt, Germany). Negative controls consisted of lymphocytes stained with the appropriate isotypes (BioLegend, San Diego, CA, USA). Measurements were performed on a flow cytometer (BD FACSVerse^TM^, BD San Diego, CA, USA). For extracellular stainings, FITC anti-CD45 (Dako, Jena, Germany), PE anti-CD8 (ImmunoTools, Friesoythe, Germany), PE anti-CD4 (ImmunoTools), FITC anti-CD14 (BioLegend, Amsterdam, The Netherlands), PE/Cy7 anti-CD163 (BioLegend), APC anti-CD169 (BioLegend), and PE anti-CD204 (BioLegend) were used. Cells were washed, resuspended in 1× PBS, and analyzed by flow cytometry on a flow cytometer (BD FACSVerse^TM^, BD Pharmingen). For each sample, 50,000 events (blood) or 1,000,000 events (PBMCs) were measured. Data analysis was performed using FlowJo^TM^ Version 10.6.1.

### Clotting Assays

All clotting times were measured using an Amelung coagulometer. For blood clotting time, 480 μL of fresh citrated human blood was incubated 1:1 with HEPES (control) or streptococcal species with indicated CFU/mL or pneumolysin concentrations for indicated time at 37°C. One hundred microliter of the blood samples was recalcified, and the clotting time was determined.

For clotting of PBMCs, 100 μL of plasma was recalcified, and 1 × 10^6^ PBMCs in a volume of 100 μL PBS were added. Time until clot formation was determined.

Clotting times in plasma after pneumolysin incubation was measured by incubating pneumolysin at different concentrations with plasma for 30 min followed by the addition of equal amounts of Dapptin (for activated partial thromboplastin time [aPTT]), containing silica, sulfatide, and phospholipids (Technoclone) for 60 s at 37°C. Clotting was initiated by the addition of 25 mM CaCl_2_. For the prothrombin time (PT) assay or thrombin clotting time (TCT), clotting was initiated by the addition of Technoplastin HIS (PT reagent, Technoclone) or thrombin reagent (Technoclone).

For clotting time evaluation after incubation of bacteria, all strains were set to a concentration of 1 × 10^10^ CFU/mL in PBS. One Hundred microliter bacterial solution was incubated with 100 μL plasma for 30 min at 37°C. PBS instead of bacteria was used for the control. Bacteria were removed by centrifugation, and clotting times (aPTT, PT, TCT) were determined in the supernatant.

To test the procoagulant effect of MVs, 50 μL MVs were incubated with pneumolysin for 20 min at 37°C. Then 100 μL recalcified plasma was added, and time until clot formation measured.

### Chromogenic Substrate Assay

Pooled diluted plasma (1:10 in 15 mM HEPES) was incubated with pneumolysin at different concentration. In the positive control, Dapptin was added, and in the negative control, water was added. One millimolar of the chromogenic substrate S-2302 (for PK/FXIIa activity, Chromogenix) was added, and the absorbance at 405 nM was measured at 37°C over a period of 120 min. No endogenous proteolytic activity was detected in the absence of plasma.

Contact activation at the bacterial surface has been measured as described before [30]. Briefly, pneumococcal cultures were set to 1 × 10^10^ CFU/mL in 15 mM HEPES, and 100 μL of the suspension was added to equal amounts of normal plasma or HEPES as the control. After incubation for 30 min at 37°C, bacteria were washed three times, resuspended in 300 μL HEPES, and supplemented with 100 μL of the chromogenic substrate S-2302 (4 mM, Haemochrom Diagnostica, Germany). After incubation for 60 min at 37°C, cells were removed by centrifugation, and absorbance was measured at 405 nM.

Measurement of contact system activation by bacterial supernatants was performed by incubation of 100 μL supernatant from overnight cultures together with 100 μL of plasma and chromogenic substrate S-2302 (1 mM). Plasma mixed with medium served as the control. Absorbance was determined at 405 nM over 120 min in the SpectraMax.

### Plasma Protein Adsorption and Western Blot Analysis

For sampling, pneumococcal cultures were set to 1 × 10^10^ CFU/mL, mixed with an equal amount of human normal plasma, and incubated at 37°C for 30 min with shaking (600 rpm). Incubation of plasma with PBS, bacteria with PBS, and plasma with DAPTTIN served as controls. After centrifugation, bacterial pellets were washed 3 times (6,800 *g*, 5 min) with PBS, resuspended in 100 μL glycine (0.1 M), and incubated at room temperature for another 10 min. The pH value of supernatants from subsequent centrifugation (12,000 *g*, 5 min) was neutralized by the addition of 20 mL Tris-HCl (1 M, pH = 8.4), and 100 μL of the suspensions was mixed with 20 μL SDS sample buffer (5×). Sampling was performed on 3 different days. SDS-PAGE was performed as described earlier [31]. Following SDS-PAGE, separated proteins from the eluates were transferred onto nitrocellulose membranes. Western blot analyses were performed with antihuman HK (Affinity Biologicals), antihuman PK (Affinity Biologicals), antihuman fibrinogen (alpha chain, Santa Cruz), antihuman FVII (Affinity Biologicals), or anti-pneumolysin (Davids Biotechnologie GmbH). Blots were incubated with secondary fluorophore-labeled antibodies (LI-COR) and imaged using an Odyssey Imager (LI-COR).

### Western Blot Overlay

To detect an interaction of plasma proteins with soluble pneumolysin, the plasma proteins (2 μg/lane), following SDS-PAGE, were transferred onto nitrocellulose membranes. The membranes were blocked, followed by incubation for 1 h with pneumolysin (10 μg/mL). After washing, the blot was incubated with the anti-pneumolysin antibody. Blots were incubated with secondary fluorophore-labeled antibodies (LI-COR) and imaged using an Odyssey Imager (LI-COR).

### Surface Plasmon Resonance

The interactions between soluble HK, fibrinogen, PK and FXII (as analytes), and pneumolysin (as ligand) were analyzed with a BIAcore 3000 system (Biosensor, La Jolla, CA, USA) using CM5 sensor chips at 25°C in HBS-EP as running buffer. Pneumolysin was immobilized on a flow cell surface of the chip to a density of 1,031 response units using standard amine-coupling chemistry and the software tool “Application Wizard-Surface Preparation” (BIAcore 3000 Instrument Handbook). The analyte-ligand complex was allowed to associate and dissociate for 3 and 5 min, respectively, with background subtraction using a flow cell that was subjected to the coupling reaction but without protein, as reference surface. For concentration series, PK and HK were tested at 25, 50, 100, and 200 nM and FXII at 100, 200, 300, and 400 nM. The surface was regenerated with a 15-s injection of 50 mM NaOH and 5-min buffer flow at the end of each binding cycle. The data from the BIAcore sensorgrams were fitted locally, using the one-step biomolecular association reaction model (1:1 Langmuir binding).

### Fluorescence Staining and Quantification

MVs were labeled with the red-fluorescence aliphatic chromophore PKH26 dye (Sigma), which intercalate into lipid bilayers, as described before [29]. As a control, PBS was treated equally and used as the blank. Labeled MVs or blank were mixed with indicated concentrations of pneumolysin and incubated for 20 min at 37°C. After incubation, fluorescence was quantified at 551/567 nM in a SpectraMax plate reader (Molecular Devices, Sunnyvale, CA, USA).

### Scanning Electron Microscopy

MVs were treated with pneumolysin for 20 min at 37°C and fixed with 2.5% glutaraldehyde for 4 h. Samples were pelleted by centrifugation at 4°C, washed 3 times with PBS, and fixed on formvar-coated grids with a carbon film (Plano). Samples were analyzed with a Quattro S scanning electron microscope (Thermo Fisher).

### Ethics Approval Statement

The protocol for the collection of human blood was approved by the Ethics Committee at the medical faculty of the University of Rostock (Ethics Committee vote: A 2014-0131). The experiments were conducted in accordance with the ICH-GCP guidelines. Informed written consent was obtained from all subjects.

### Statistics

All values are reported as mean ± SD. Differences between controls and treated samples were determined by using the unpaired *t*-test. If normality failed, the nonparametric Mann-Whitney U-test was applied. In case of multiple comparisons, one- or two-way ANOVA on ranks was applied, with Dunnett's posttest (to compare with the control) or the Sidak's posstest (to compare preselected pairs of columns). If normality failed, the Kruskal-Wallis test with Dunn's posttest was used. Statistical evaluation was performed using GraphPad Prism software, version 8.43 (GraphPad Software, San Diego, CA, USA). The criterion for significance was taken to be *p* < 0.05.

## Results

### Procoagulant Activity of Blood after Incubation with Bacteria

**Fig. 1:**
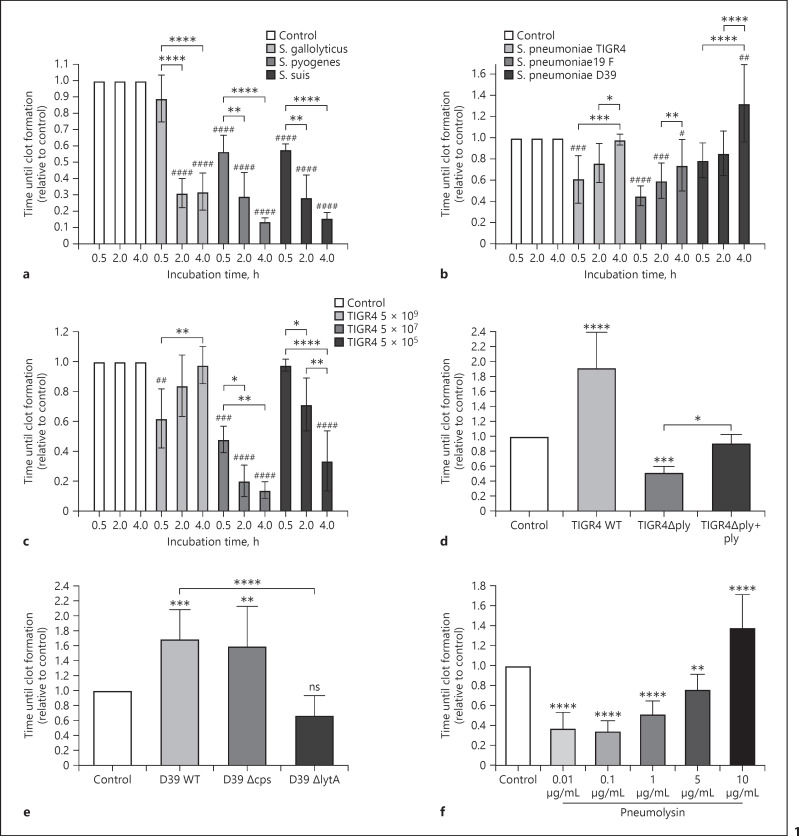
Procoagulant activity of human blood in response to different bacterial strains and pneumolysin. Human blood was incubated with indicated CFU bacteria or pneumolysin for 0.5, 2, or 4 h at 37°C. Blood samples were recalcified, and the time until a clot forms was determined in a coagulometer. **a, b** Streptococcal strains *S. pyogenes, S. suis, S. gallolyticus*, or *S. pneumoniae* (19F, TIGR4, D39) were tested with a concentration of 5 × 10^9^ CFU/mL blood. **c** *S. pneumoniae* TIGR4 WT was tested with different concentrations (5 × 10^5^, 5 × 10^7^, and 5 × 10^9^ CFU/mL blood). **d** WT *S. pneumoniae* strain TIGR4 (TIGR4 WT) or its *∆ply* deletion mutant was used with a concentration of 5 × 10^9^ CFU/mL blood and incubated for 4 h. In addition, the *∆ply* strain was incubated in the presence of recombinant pneumolysin (1 μg/mL, *∆ply* + Ply). **e** WT *S. pneumoniae* strain D39 (D39 WT) or its capsule deletion mutant strain (*∆cps*) or its autolysin deletion mutant (*∆lytA*) was used with a concentration of 5 × 10^9^ CFU/mL blood and incubated for 4 h. **f** Pneumolysin was incubated with indicated concentrations in blood for 4 h. The values express coagulation times relative to the coagulation time evoked by buffer-treated blood (control). Values below 1 indicate increased procoagulant activity and values above 1 a delayed time until clot formation. Bars represent means and SD from three–four different donors. Asterisks indicate statistically significant differences as compared to the control, if not otherwise indicated. Significance was determined by one- or two-way ANOVA with Dunnett's or Sidak's posttest. *p < 0.05; **p < 0.005; ***p < 0.001; ****p < 0.0001.

Incubation of human blood with bacteria or bacterial components usually triggers procoagulant activity, verifiable by a significant shortening of the blood coagulation time [28, 32, 33, 34]. To test whether pneumococci and other streptococci induce procoagulant activity, bacteria were set to 5 × 10^9^ CFU/mL in blood and incubated for 0.5, 2, and 4 h. One hundred microliter of the sample was taken at indicated time points, recalcified, and the time until formation of a blood clot was measured. Coagulation times were normalized to the control samples (without bacteria) of the corresponding donor. Incubation of blood with *Streptococcus suis, Streptococcus pyogenes*, or *Streptococcus gallolyticus* shortened blood coagulation times significantly, compared to controls (Fig. 1a), thus showing a significant increase of procoagulant activity in the blood sample over time. Intriguingly, after incubation with different pneumococcal strains from either invasive (*S. pneumoniae* D39, TIGR4 [35]) or noninvasive infections (*S. pneumoniae* 19F [20]), an opposite coagulation pattern was observed (Fig. 1b). Here, the blood coagulation times were also significant shorter for TIGR4 and 19F after 30 min of incubation, compared to the control, showing activation of coagulation in the blood sample. However, with longer incubation time, the blood coagulation time extended significantly in the same blood sample, when compared to the earlier time point. This implicates that the coagulation-promoting activity in the blood sample decreases after longer incubation with pneumocci (Fig. 1b). This was in contrast to the results obtained with the other investigated streptococci. If less pneumococci were used (TIGR4, 10^7^ and 10^5^ CFU/mL), the coagulation pattern was comparable with the other streptococcal strains (Fig. 1c); thus, higher concentrations of pneumococci are necessary to induce the decline of procoagulant activity.

Additionally, when using 10^9^ CFU/mL pneumococci, blackening of the blood was observed with longer incubation time, and lysis of blood cells was microscopically detected in blood smears (not shown). *S. pneumoniae* produces a potent toxin, pneumolysin, that is released when the pneumococci undergo autolysis, which might be the main reason for the declining procoagulant activity of the blood over time. To prove this, we used different mutant strains − deficient in pneumolysin (*∆ply*), autolysin (*∆lytA*), or the capsule (*∆cps*) − and incubated them in blood for 4 h before measuring the time until clot formation. Indeed, when we used the pneumolysin mutant (*∆ply*), a significant shortened blood coagulation time was measured after 4 h, compared to the control (Fig. 1d). Moreover, if the *∆ply* strain was complemented with purified pneumolysin (1 μg/mL), the coagulation time was significantly longer, compared to the *∆ply* mutant strain (Fig. 1d). When the *∆lytA* mutant was used, the blood coagulation time was significantly shortened, compared to the D39 WT (Fig. 1e). Moreover, incubation with the wild-type (WT) strains TIGR4 and D39 significantly prolonged the coagulation time after 4 h, compared to the control samples (Fig. 1d, e). The lack of the capsular polysaccharide in the tested strain (*∆cps*) did not change the clotting pattern, as also a delayed blood coagulation time was measured (Fig. 1e).

The data indicate that a massive release of pneumolysin − due to a higher bacterial number − is responsible for slowing blood coagulation over time. We therefore incubated blood with different concentrations of recombinant purified pneumolysin for 4 h and measured the blood coagulation time. Figure 1f shows that at pneumolysin concentrations from 0.01 μg/mL, the coagulation time was significantly shortened, compared to the control. The pneumolysin concentration causing procoagulant activity (0.01 μg/mL) corresponds to the concentration causing hemolysis of erythrocytes (online suppl. Fig. 1a; for all online suppl. material, see www.karger.com/doi/10.1159/000525479). Importantly, increasing pneumolysin concentrations resulted in increasing blood coagulation times and thereby a decline of procoagulant activity (Fig. 1f). The same effect was observed with increasing numbers of pneumolysin-producing bacteria (see Fig. 1c). Analysis of T cells and monocytes by FACS revealed a decrease of CD14^+^ cells with pneumolysin concentration from 1 μg/mL; however, CD4^+^ and CD8^+^ T cells were reduced by higher concentration (5 μg/mL, see online suppl. Fig. 2a, b). Examination of blood smears from the samples showed that all cells were completely lysed at a pneumolysin concentration of 10 μg/mL (online suppl. Fig. 1c), the same concentration that caused a significant delay of the blood coagulation time (Fig. 1f).

To assess the concentration of pneumolysin in TIGR4 WT cultures, bacterial supernatants were investigated by hemolysis-assays, SDS-PAGE and Western blotting (online suppl. Fig. 1). When using bacterial supernatants from 5 × 10^7^ or 5 × 10^9^ CFU pneumococci, a complete hemolysis of erythrocytes was observed (online suppl. Fig. 1a, b). The intensities of the pneumolysin protein bands from the supernatant of 5 × 10^9^ CFU/mL TIGR4 correspond to 6 (±2.4) µg/mL pneumolysin after 4 h of incubation (online suppl. Fig. 1c).

Taken together, in the presence of high numbers of pneumococci, but not other tested streptococcal species, blood coagulation time was severely delayed. This effect arises if high amounts of pneumolysin are released.

### Autolysin, but Not Pneumolysin, Induced Procoagulant Activity of PBMCs

**Fig. 2:**
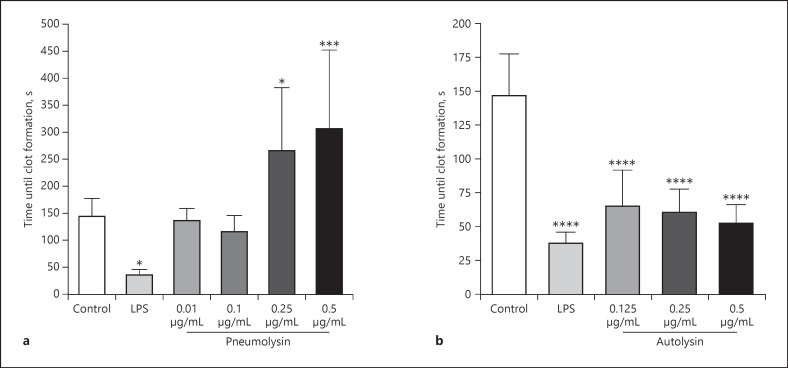
Procoagulant activity of human PBMCs in the presence of recombinant pneumolysin (**a**) or autolysin (**b**). PBMCs were isolated from human blood, and 1 × 10^6^ cells were incubated with pneumolysin or autolysin at indicated concentrations or 1 μg/mL LPS for 20 h at 37°C. Buffer-incubated cells were used as the control. Cell suspensions were then added to pre-warmed citrated human plasma and recalcified with 25 mM CaCl_2_, and the time to form a clot was measured. Values represent the mean ± SD of four different donors, each done in duplicate. Asterisks indicate statistically significant differences as compared to the control. Significance was determined one-way ANOVA with Dunnett's posttest *p < 0.05; ***p < 0.001, ****p < 0.0001.

It is known that treatment of blood or peripheral blood mononuclear cells (PBMCs) with LPS and other pathogen-associated molecular patterns (PAMPs) induces procoagulant activity of monocytes via increased tissue factor production of these cells [32, 36]. Based on our findings with whole blood, we tested whether the interaction between pneumolysin or autolysin and PBMCs is responsible for the induction of procoagulant activity in human blood. To that end, human PBMCs were treated with different concentrations of pneumolysin or autolysin for 20 h. Cells were then added to fresh recalcified human plasma, and the time to form a clot was measured. In contrast to whole blood experiments, treatment of PBMCs with lower concentrations of pneumolysin (from 0.25 μg/mL) caused a significant delay of clot formation, compared to the control (Fig. 2a). This was caused by the cytotoxic effect of pneumolysin on the cells as incubation with 0.5 μg/mL pneumolysin for 20 h reduced the total cell count by over 99% and CD14^+^ cells by over 95% (see online suppl. Fig. 3).

In contrast, treatment of PBMCs with LPS (1 μg/mL) or autolysin accelerated clot formation significantly (Fig. 2a, b). These findings show that autolysin − similarly to LPS and other PAMPs − increased the procoagulant activity of PBMCs. Thus, at sublytic concentrations pneumolysin did not accelerate PBMC-induced clot formation significantly, and if lytic concentrations were used (from 0.25 μg/mL), initiation of coagulation by PBMCs was significantly delayed due to cell lysis.

### Autolysin Induces the Release of Pro- and Anti-Inflammatory Cytokines in PBMCs

**Fig. 3:**
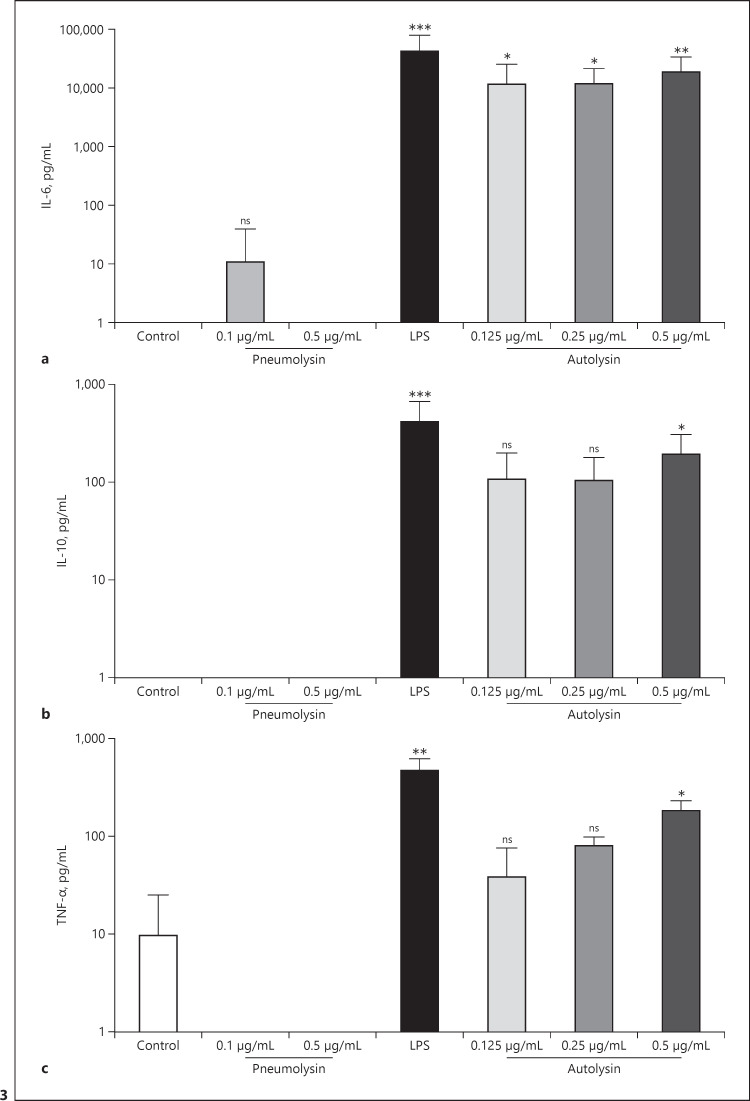
Cytokine release from human PBMCs in the presence of recombinant pneumolysin or autolysin. PBMCs were isolated from human blood, and 1 × 10^6^ cells were incubated with pneumolysin or autolysin at indicated concentrations or 1 μg/mL LPS for 20 h at 37°C. Buffer was used as the control. IL-6 (**a**), IL-10 (**b**), and TNF-α (**c**) were determined in the supernatant by ELISA. Values represent the mean ± SD of four different donors, each done in duplicates. Asterisks indicate statistically significant differences as compared to the control. Significance was determined by the Kruskal-Wallis test with Dunn's posttest *p < 0.05; **p < 0.005; ***p < 0.001.

It has been shown that the induction of procoagulant activity in blood or PBMCs by LPS and other PAMPs is associated with the release of pro- and anti-inflammatory cytokines [27, 37, 38]. We therefore measured IL-6, IL-10, and TNFα in the supernatant of PBMCs, treated with pneumolysin or autolysin. Only autolysin treated PBMCs released all tested cytokines with significantly higher concentrations, compared to control samples (Fig. 3a-c).

### Adsorption of Coagulation and Contact System Proteins at the Pneumococcal Surface Is Dependent on Pneumolysin

**Fig. 4:**
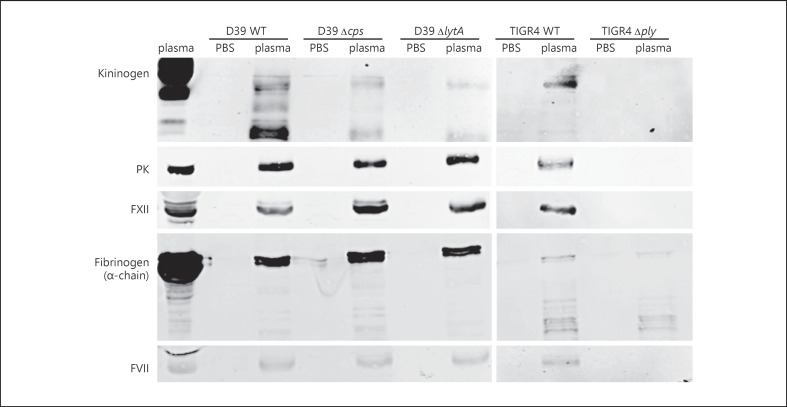
Adsorption of plasma proteins on the surface of pneumococci and mutant strains. Bacteria (1 × 10^10^ CFU/mL) were incubated with human plasma or PBS for 30 min at 37°C. After washing, bacteria-bound proteins were eluted with a glycine buffer (eluate). Activated plasma (plasma activated by DAPPTIN) and eluates were separated on SDS-PAGE, transferred to a membrane, and immunostained with polyclonal antibodies against HK, PK, FXII, fibrinogen alpha chain, or FVII. Experiments were done 3 times with pooled plasma, and one representative picture is shown.

*S. pyogenes* and *S. gallolyticus* adsorb and bind coagulation and contact factors from plasma [25, 39, 40, 41], thereby impairing coagulation and prolonging the clotting times in plasma. We therefore tested TIGR4 WT and the isogenic *∆ply* strain, as well as D39 WT and its D39-*∆csp* and D39-*∆lytA* strains for adsorption of some coagulation and contact system proteins at the bacterial surface. Bacteria were incubated in plasma for 30 min, washed, and adsorbed proteins were eluted from the surface. As controls, bacteria were incubated in PBS buffer. The eluates were investigated by Western blot analysis for the containment of fibrinogen, HK, PK, FXII, and factor VII (FVII). All plasma proteins were detected in eluates from the WT D39 and TIGR4 (Fig. 4). Moreover, the eluates from deletion mutants *∆cps* and *∆lytA* also contained fibrinogen, HK, PK, FXII, and FVII. In contrast, in eluates from the *∆ply* mutant strain, only fibrinogen was detected (Fig. 4). Taken together, pneumococci adsorb different plasma proteins, but the adsorption of HK, PK, FXII, and FVII seems to be dependent on pneumolysin production as the eluates of the *∆ply* mutant strain did not contain these proteins. Adsorption of fibrinogen however was independent from pneumolysin production.

### Interference of Pneumococci with Coagulation and Contact System Activation

We next tested whether adsorption of coagulation and contact system proteins at the pneumococcal surface influence plasma clotting times. Bacterial strains were incubated with human plasma for 30 min and then removed by centrifugation. PT, TCT, and aPTT were determined in the supernatants. These parameters are commonly used in clinical practice for the global assessment of plasma coagulation. The PT evaluates the extrinsic coagulation pathway, whereby FXII activation is the mechanistic basis for the aPTT. For the TCT test, addition of thrombin allows formation of a stable clot. If one component of the coagulation pathway is not present in sufficient quantity, after incubation of the bacteria in plasma, a significant prolongation of the clotting times can be measured [25, 41, 42, 43].

**Fig. 5:**
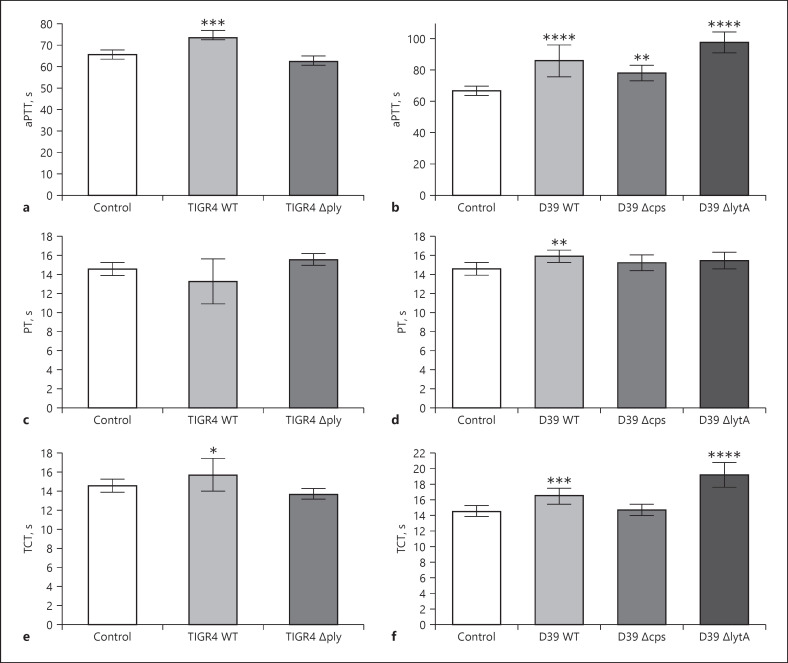
Clotting of plasma after incubation with pneumococcal strains. 2 × 10^10^ CFU/mL bacteria were incubated in human plasma for 30 min at 37°C. Plasma incubated with buffer was used as the control. Bacteria were removed, and the aPTT (**a, b**) or the PT (**c, d**) or the TCT (**e, f**) of the supernatant was determined in a coagulometer. Data represent mean values and standard deviation, whereas mean values result from three independent biological measurements. Significance values calculated in reference to the control using the one-way ANOVA with Dunnet's posttest test. *p < 0.05, **p < 0.01, ***p < 0.001, ****p < 0.0001.

Significantly prolonged aPTT (Fig. 5a, b) values were observed for all bacterial strains − except *∆ply* − as compared to plasma samples incubated with buffer alone. Only D39 WT prolonged the PT time slightly, however significantly, compared to the samples without bacteria (Fig. 5d). The TCT was also slightly, but significantly, prolonged after incubation of both WT and the *∆lytA* strains (Fig. 5e, f) but remained unchanged after incubation with *∆ply* (Fig. 5e) or *∆cps* (Fig. 5f). Thus, this result strongly supports our Western blot analyses (Fig. 4), whereby all strains − except *∆ply* − adsorb the contact factors HK, PK, and FXII, as well as the coagulation factor FVII, on their surface, which ultimately affected clotting times.

We and others have shown that binding of contact factors at the bacterial surface can trigger activation of the contact system [25, 31, 42]. However, when we investigated activity of FXIIa/PK at the pneumococcal surface, or with pneumococcal supernatants, no activity could be detected (online suppl. Fig. 4a, b).

### Interaction of Pneumolysin with Coagulation and Contact Factors

**Fig. 6:**
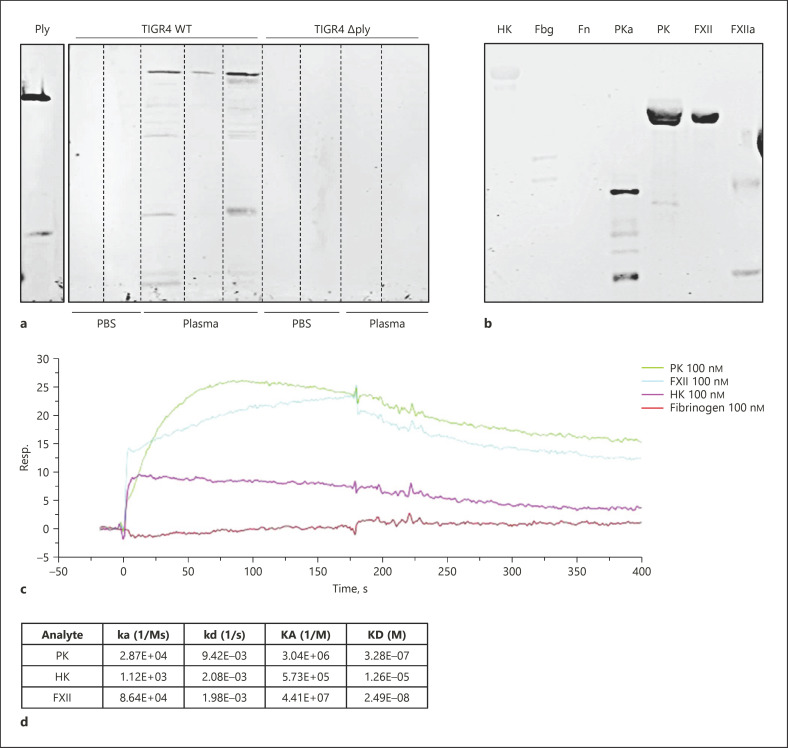
Interaction of pneumolysin with coagulation and contact system factors. **a** Bacteria (1 × 10^10^ CFU/mL) were incubated with human plasma or PBS for 30 min at 37°C. After washing, bacteria-bound proteins were eluted with a glycine buffer. Eluted proteins were separated on SDS-PAGE, transferred to a membrane, and immunostained with a polyclonal antibody against pneumolysin. **b** Binding of pneumolysin to immobilized plasma proteins, investigated by Western blot. Each plasma protein was used at a concentration of 2 μg/lane. **c** Binding properties of pneumolysin measured by SPR. Pneumolysin was coupled to a sensor chip and subjected to injections with 100 nM PK, FXII, HK, or Fbg. **d** Serial dilutions of PK, HK, or FXII were injected to the pneumolysin-coated sensor chip, and the kinetic and affinity parameters of this interaction are listed. SPR, surface plasmon resonance; HK, high-molecular-weight kininogen; Fbg, fibrinogen; Fn, fibronectin; PK, plasma kallikrein; PKa, activated PK; FXII, factor XII; FXIIa, activated FXII.

The absence of pneumolysin abolished adsorption of some plasma proteins at the bacterial surface (see Fig. 4). It has been shown that pneumolysin is probably also attached to the bacterial cell surface [44]. Thus, we assumed that bacteria-associated pneumolysin interacts with plasma proteins. We checked first whether the bacterial eluates from the plasma adsorption experiments contain pneumolysin. As expected, pneumolysin was detected exclusively in eluates from TIGR4 WT (Fig. 6a). However, pneumolysin could only be detected by this method, when the TIGR4 WT was incubated in plasma, but not after incubation in PBS (Fig. 6a). This implicates that pneumolysin potentially reassociates to the pneumococcal surface in the presence of plasma.

We further investigated whether recombinant pneumolysin binds contact and coagulation factors. In a first step, purified plasma proteins of interest were subjected to SDS-PAGE and Western blotting, followed by exposure to pneumolysin and detection with a pneumolysin antibody. As depicted in Figure 6b, pneumolysin shows strong reactivity with the blotted contact factors PK and FXII, as well as with their activated forms (activated PK and FXIIa). In contrast, little or no reactivity of pneumolysin with blotted HK, fibrinogen, or fibronectin was detected (Fig. 6b). Binding properties were further investigated by surface plasmon resonance. Sensor chips were coated with pneumolysin and probed with PK, FXII, HK, or fibrinogen. Consistent with the Western blot analysis, FXII, PK, and HK, but not fibrinogen, bound to immobilized pneumolysin (Fig. 6c). The binding affinity (estimated KDs) between pneumolysin and FXII or PK was in the median nanomolar range and for pneumolysin-HK interaction in the lower micromolar range (Fig. 6d). We further investigated whether binding of pneumolysin to FXII or PK triggers activation of the zymogens; however, pneumolysin neither activated FXII or PK in plasma (online suppl. Fig. 4c) nor did HK degradation occur in plasma, to which pneumolysin (up to 10 μg/mL) was added (online suppl. Fig. 4d).

### Interference of Pneumolysin with Activation of Coagulation

We next tested whether pneumolysin might directly interfere with plasma coagulation and incubated it at different concentrations in plasma for 30 min at 37°C. Clotting times (aPTT, PT, and TCT) were determined after incubation; however, pneumolysin had no effect on clotting times after this procedure (data not shown).

**Fig. 7:**
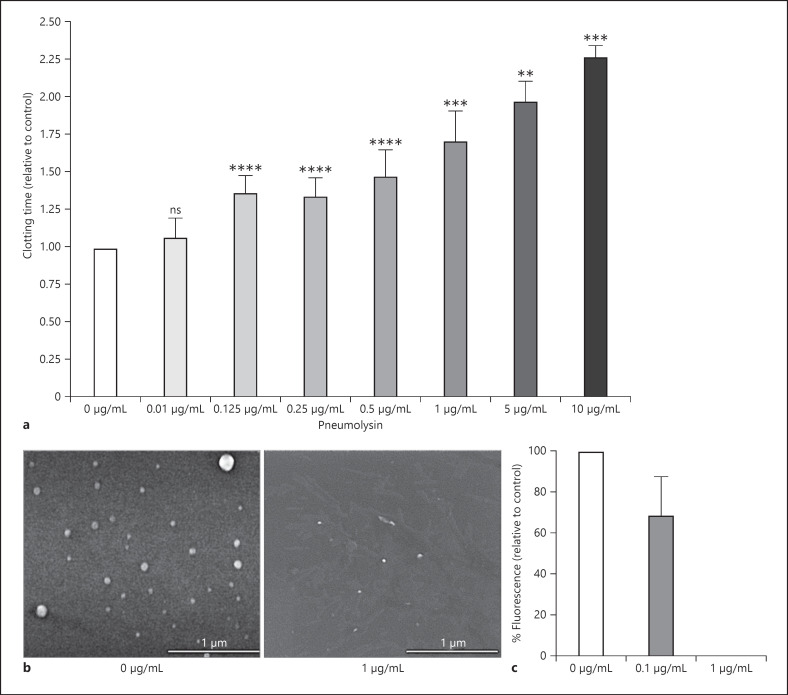
PBMC-derived procoagulant MVs after incubation with pneumolysin. PBMCs were stimulated with LPS for 24 h, and MVs were puriﬁed from the supernatant as described in methods. **a** MVs were incubated with indicated concentrations of pneumolysin or buffer (0 μg/mL) for 20 min at 37°C. MVs were then added to re-calciﬁed plasma, and the time for clot formation was determined. Clotting times were normalized for each donor in relation to the control (0 μg/mL), which value was set to 1. **b** SEM of procoagulant MVs treated with buffer (0 μg/mL) or 1 μg/mL pneumolysin (scale bar, 1 μm). **c** MVs were stained with PKH26 and incubated with indicated pneumolysin concentrations for 20 min at 37°C. Fluorescence was detected in SpectraMax. Absorbance was normalized for each donor in relation to the MV control (0 μg/mL), which value was set 100%. Values represent the mean ± SD of MVs from four different donors. Significance values calculated in reference to the control using the one-way ANOVA with Dunnet's posttest test **p < 0.005, ***p < 0.0002, ****p < 0.0001. SEM, scanning electron microscopy.

During sepsis, procoagulant tissue factor-containing MVs released by monocytes or activated endothelium [45] trigger procoagulant activity and thrombosis. It has been also postulated that tissue factor-containing MVs play a key role in normal coagulation [46]. We tested whether pneumolysin interferes with the procoagulant activity of PBMC-derived MVs. Procoagulant MVs were derived from activated PBMCs as described before [28, 29] and were treated with different concentrations of pneumolysin for 20 min at 37°C. After treatment, MVs were added to recalcified plasma, and the clotting time was measured. Addition of buffer-treated MVs to recalcified plasma (Fig. 7a) induced coagulation, whereby addition of buffer alone did not lead to formation of a plasma clot within 995 s (not shown). If MVs were treated with increasing concentration of pneumolysin, the MV-induced clotting time was significantly prolonged (Fig. 7a), compared to the control (buffer-treated MVs).

Scanning electron microscopy analysis suggests that the number of MVs is drastically reduced after incubation with 1 μg/mL pneumolysin for 30 min (Fig. 7b). To quantify MVs, PKH26-stained MVs were treated with different concentrations of pneumolysin, and fluorescence was detected in SpectraMax. Fluorescence was reduced after treatment with 0.1 μg/mL pneumolysin and completely absent if 1 μg/mL pneumolysin was incubated for 30 min (Fig. 7c). Taken together, these data indicate that procoagulant MVs lose their procoagulant properties after treatment with pneumolysin.

## Discussion

Here, we show that the pore-forming toxin pneumolysin − when released in large amounts by pneumococci − impaired coagulation of blood, a physiological host defense mechanism to prevent bleeding and contain bacteria at the local place of infection [47]. This effect was observed with higher numbers of bacteria (10^9^ CFU/mL), which contain around 6 μg/mL pneumolysin in the supernatant, according to the present and a previous study [12]. In different mice models of pneumococcal pneumonia, up to 10^9^ CFU/mL bacteria were detected in the lungs or in the bronchoalveolar lavage (BAL) [10, 48, 49, 50]. Concentrations of bacteria or pneumolysin in the lung of patients have not been determined yet. In an experimental pneumococcal pneumonia mouse model, sublytic pneumolysin concentrations were determined in the BAL, whereby a high number of bacteria (up to 10^9^ CFU) were counted [10]. However, pneumolysin concentrations in the cerebral fluid of patients with pneumococcal meningitis were much higher, with 30 μg/mL in survivors and 75 μg/mL in non-survivors [51], whereas in animal models of pneumococcal meningitis, the determined pneumolysin concentration was 0.02 μg/mL [52]. Thus, it remains difficult to transfer animal experimental data to humans and draw conclusions about in vivo concentrations of pneumolysin. We observed impaired coagulation of whole blood with 10 μg/mL pneumolysin. If coagulation was induced by MVs or PBMCs, pneumolysin concentration of 0.125 or 0.25 μg/mL is sufficient to prolong the clotting time. Moreover, diminution of the procoagulant activity by pneumolysin also depends on the incubation time because even with low pneumolysin concentrations, cells are destroyed after a longer exposure time [12]. Another study demonstrated that T lymphocytes and monocytes will be lysed by 0.15 μg pneumolysin within 12 min [53]; therefore, probably much lower concentrations of pneumolysin than we used here in vitro can lead to cell and MV damage in vivo.

The increased procoagulant activity of blood − due to infection or inflammation − is mediated by the production of tissue factor from monocytes that is released in form of tissue factor-containing MVs [54, 55, 56, 57, 58, 59]. MVs are membrane-bound particles that are less than 0.5 μm in size and produced by nearly all cell types (monocytes, platelets, endothelial cells) following an inflammatory stimulus. The function and regulation of tissue factor activity in the lung is complex, involving enhanced synthesis by endothelial cells and the subsequent release of tissue factor-containing procoagulant MVs [60]. Tissue factor is important for a normal lung function as low-tissue-factor mice have chronic hemorrhage and increased lung permeability [61]. Moreover, tissue factor-dependent activation of coagulation in the lung seems to be essential to prevent lung damage in infection and inflammatory conditions [16, 17]. These findings from animal studies were further supported by two clinical trials using recombinant tissue factor pathway inhibitor (TFPI), to block tissue factor activity. Neither of these trials showed a clinical beneﬁt [62, 63]. Procoagulant, tissue factor-containing MVs participate actively in coagulation [64] and can contribute to the early innate host defense [28, 29]. High levels of leukocyte-derived MVs in the BAL were associated with a better outcome in ARDS [65]. It has been reported that procoagulant MVs − due to their bigger surface − have a 50- to 100-fold higher specific procoagulant activity than activated platelets [66]. Thus, we suggest that the destruction of MVs by pneumolysin might severely impair hemostasis and coagulation in the lung and promote hemorrhage. An intriguing study has been shown that pneumolysin induces membrane deformation and wrinkling in artificial lipid vesicles [67]; however, it remains to be investigated whether this mechanism occurs also in cell-derived MVs.

On the other hand, pneumolysin itself causes the release of MV-like structures from different cell lines, when incubated at nanomolar concentrations for a short exposure time [53, 67, 68]. This shedding of pneumolysin-containing MVs has been described as an essential process to seal damaged plasma membrane areas after pneumolysin attack [53]. Whether PBMCs produce MVs after pneumolysin attack and whether such MVs are procoagulant remains to be investigated. We did not detect increased procoagulant or proinflammatory activity of PBMCs after incubation with sublytic concentrations of pneumolysin (see Fig. 2, 3). However, incubation of whole blood with lower concentrations of pneumolysin triggered procoagulant activity, although higher pneumolysin concentrations reversed this effect (see Fig. 1f). This is not unexpected since the erythrocytes initially compensate for the lytic effect on the PBMCs in blood. Lysis of erythrocytes with release of hemoglobin might also contribute to activation of coagulation in these settings [69, 70, 71, 72, 73]. High amounts of free hemoglobin on the other hand have been shown to inhibit the contact phase of blood coagulation [74].

Autolysin alone had a procoagulant and proinflammatory effect on PBMCs, which indicates that autolysin can act as a PAMP. Our experiments with strains deficient in pneumolysin or autolysin (Fig. 1d, e) indicate that the release of autolysin in the beginning might trigger procoagulant activity; however, this procoagulant effect is reversed at later time points by the lytic effect of pneumolysin.

Additionally, pneumolysin-producing pneumococci adsorbed coagulation and contact system proteins, which consequently prolonged clotting times in plasma. In contrast, the pneumolysin-deficient mutant lacked this phenotype. This indicates that pneumolysin − at the surface of pneumococci − is responsible for the binding of plasma proteins. Although, still under debate, it was suggested that pneumolysin associates with the pneumococcal cell wall [44]. However, we were not able to detect pneumolysin in surface eluates − if plasma was absent. Our data from the plasma adsorption experiments indicate that pneumolysin reassociates to the bacterial surface, where it could be responsible for the binding of plasma proteins. Free and immobilized pneumolysin bound to the contact factors FXII, PK, and HK, which underpins this assumption. It remains to be investigated which plasma proteins support the reassociation of pneumolysin.

Binding of contact factors to the bacterial surface often induces contact activation [18]; however, this was not the case with pneumococci. It has been shown before that pneumococci do not activate the contact system [75], and also, our data show that neither the pneumococcal strains TIGR4 and D39 nor free pneumolysin were able to induce contact activation, beside the fact that they bind contact factors [21].

Thus, it seems to be more likely that pneumococci − by the release of pneumolysin − interfere with the activation of the coagulation and contact system, which might subsequently cause lung hemorrhages. It has been demonstrated that increased pneumococcal virulence was driven by the release of large quantities of pneumolysin, enabling rapid bacterial dissemination and cytotoxic damage in form of massive hemorrhage in the lung of infected animals [8]. These findings are supported through the present and previous studies, showing that high concentrations of pneumolysin destroy platelets [12] and procoagulant MVs, both main players for functioning hemostasis.

## Statement of Ethics

The protocol for the collection of human blood was approved by the Ethics Committee at the medical faculty of the University of Rostock (Ethics Committee vote: A 2014-0131). The experiments were conducted in accordance with the ICH-GCP guidelines. Informed written consent was obtained from all subjects.

## Conflict of Interest Statement

The authors declare no conflict of interest.

## Funding Sources

This work was supported by the Federal Excellence Initiative of Mecklenburg Western Pomerania and European Social Fund (ESF) Grant KoInfekt (ESF_14-BM-A55-0010_16 and ESF_14-BM-A55-0001_16) and in parts by the Deutsche Forschungsgemeinschaft (grants to SOH, project OE 547/ 4-1, and to SH, project 374031971-TRR240). Purchase of the LICOR was kindly supported by the EU-EFRE (European Funds for Regional Development) program (Registration number GHS-18-0030).

## Author Contributions

Sonja Oehmcke-Hecht conceived, designed and performed experiments, and wrote the manuscript; Claudia Maletzki and Surabhi Surabhi performed experiments; Nikolai Siemens and Sven Hammerschmidt contributed to reagents and materials and reviewed and revised the manuscript; Lisa Marie John and Sina Mariella Peter performed the experiments; Valeria Khaimov contributed analytical tools and reviewed and revised the manuscript; Bernd Kreikemeyer reviewed and revised the manuscript.

## Data Availability Statement

All data generated during this study are included in this article and its online supplementary material files. Further inquiries can be directed to the corresponding author upon reasonable request.

## Supplementary Material

Supplementary data

Supplementary data

Supplementary data

Supplementary data

## Figures and Tables

**Table 1:** Bacterial strains used in this study

Strains	Resistance	Reference
*S. pneumoniae 19F*	none	[20]
*S. pneumoniae D39*	none	NCTC 7466
*S. pneumoniae D39Δcps*	KmR	[21]
*S. pneumoniae D39ΔlytA*	KmR	[22]
*S. pneumoniae TIGR4*	none	[23]
*S. pneumoniae TIGR4Δply*	CmR	[24]
*S. gallolyticus* UCN34	None	[25]
*S. pyogenes AP1*	none	*covS* truncated clinical isolate of the M1 serotype strain 40/58 from the WHO Collaborating Centre for Reference and Research on Streptococci, Prague, Czech Republic
*S. suis*	none	[26]
