# Supplementary material for: Procoagulant Activity of Blood and Microvesicles Is Disturbed by Pneumococcal Pneumolysin, Which Interacts with Coagulation Factors
Source: J Innate Immun. 2022 Jul 15;15(1):136–52. doi: 10.1159/000525479 (PMC10643893; doi:10.1159/000525479)
Supplement: Supplementary file 1 — Supplementary data [file jin-0015-0136-s01.pdf]

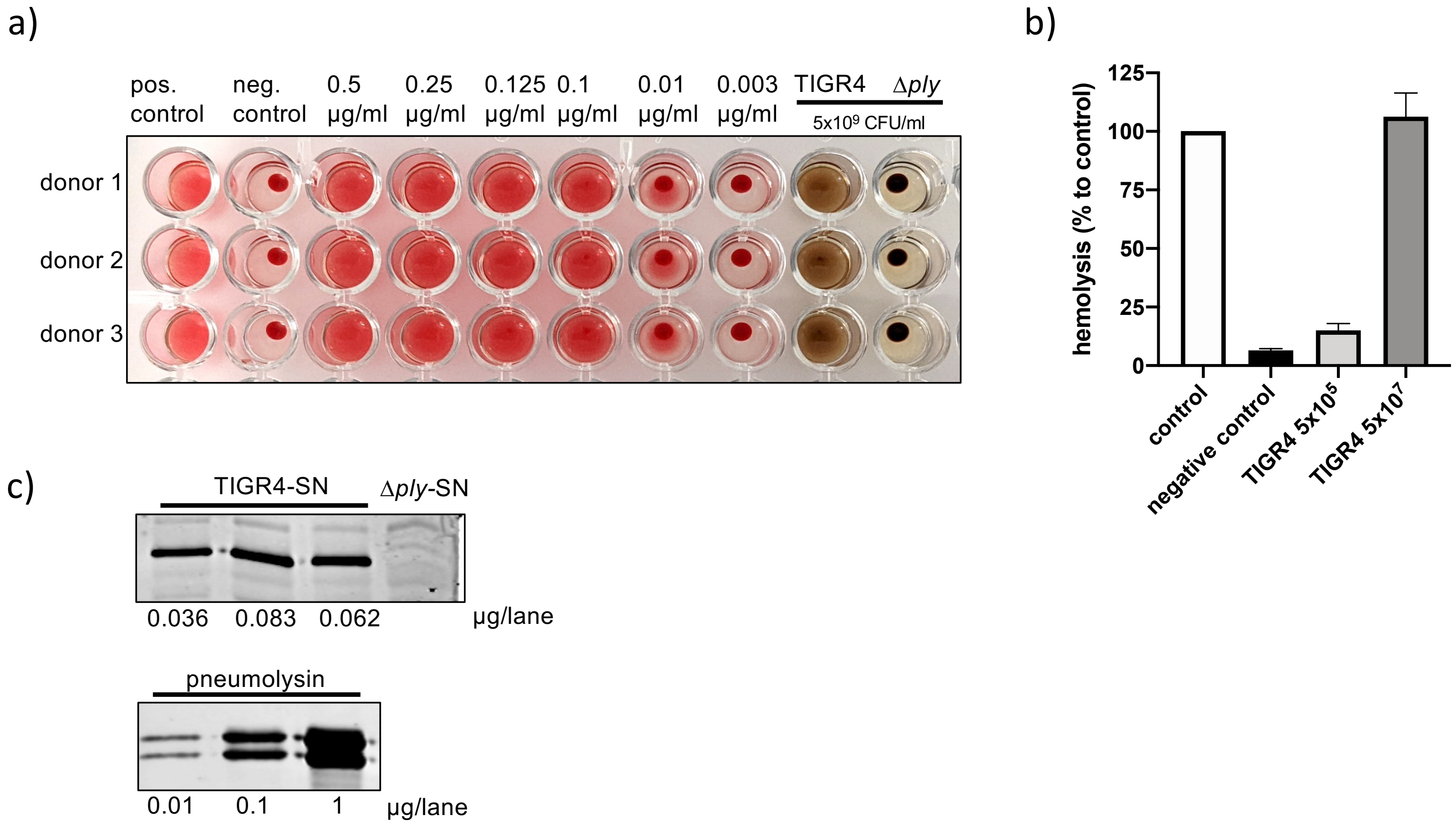

suppl. Fig. 1: Hemolytic activities of bacterial supernatants and pneumolysin and quantification of pneumolysin a) TIGR4 WT and Δ*ply* were grown until late exponential phase, set to $5x10^9$ CFU/ml in HEPES buffer and incubated for 4h at 37°C. The supernatants, or different concentrations of pneumolysin were incubated with blood from healthy human volunteers for 60 minutes at 37°C in a 96-well plate (U-bottom). After incubation, the plate was centrifuged and monitored for formation of the erythrocyte sediment.

b) TIGR4 WT was grown until late exponential phase, set to the desired CFU/ml in HEPES buffer and incubated for 4h at 37°C. The supernatants were sterile filtered and incubated with blood from healthy human volunteers for 60 minutes at 37°C. After incubation, the samples were centrifuged and the absorbance at 540 nm was quantified. Water was used as positive control and set to 100 % hemolysis.

c) TIGR4 WT and Δ*ply* supernatants (SN) from $5x10^9$ CFU/ml in HEPES buffer, incubated for 4 h at 37°C were immunoblotted and pneumolysin was detected using the antibody (see method-section). For calculation of the pneumolysin amount in the supernatants a standard curve of serially diluted recombinant pneumolysin was used.
