# Supplementary material for: Procoagulant Activity of Blood and Microvesicles Is Disturbed by Pneumococcal Pneumolysin, Which Interacts with Coagulation Factors
Source: J Innate Immun. 2022 Jul 15;15(1):136–52. doi: 10.1159/000525479 (PMC10643893; doi:10.1159/000525479)
Supplement: Supplementary file 2 — Supplementary data [file jin-0015-0136-s02.pdf]

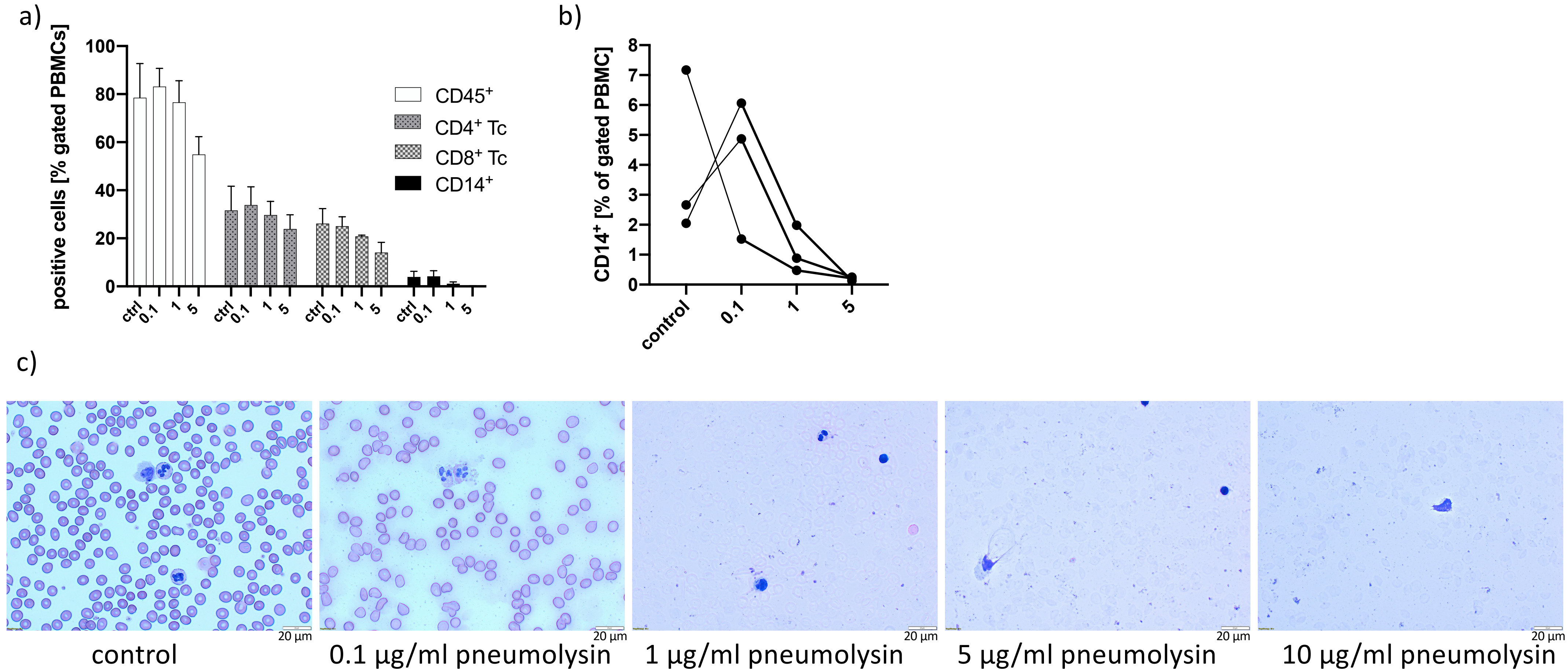

suppl. Fig. 2. Analysis of leukocyte-marker in human blood after incubation with pneumolysin. Human blood was incubated with pneumolysin-concentration from 0.1, 1.0, and 5.0 µg/ml for 4h at 37°C and analyzed by FACs (a, b) or by light microscopy (c). For FACS, whole blood was stained with specific monoclonal antibodies as stated in material and methods. Erythrocytes were lysed using erythrocyte lysis buffer and leukocytes were conducted to flow cytometry. Given is the number of % immune cells resulting from 50,000 events measured on a flow cytometer (BDFACSVerse™). a) Distribution of T-cells ($CD4^+$ and $CD8^+$ Tc) and monocytes ($CD14^+$ cells) in the gated PBMC fraction. b) $CD14^+$ monocytes decrease after treatment with 1 µg/ml pneumolysin. c) Blood smears were stained with Giemsa and investigated by light microscopy.
