# Supplementary material for: Procoagulant Activity of Blood and Microvesicles Is Disturbed by Pneumococcal Pneumolysin, Which Interacts with Coagulation Factors
Source: J Innate Immun. 2022 Jul 15;15(1):136–52. doi: 10.1159/000525479 (PMC10643893; doi:10.1159/000525479)
Supplement: Supplementary file 3 — Supplementary data [file jin-0015-0136-s03.pdf]

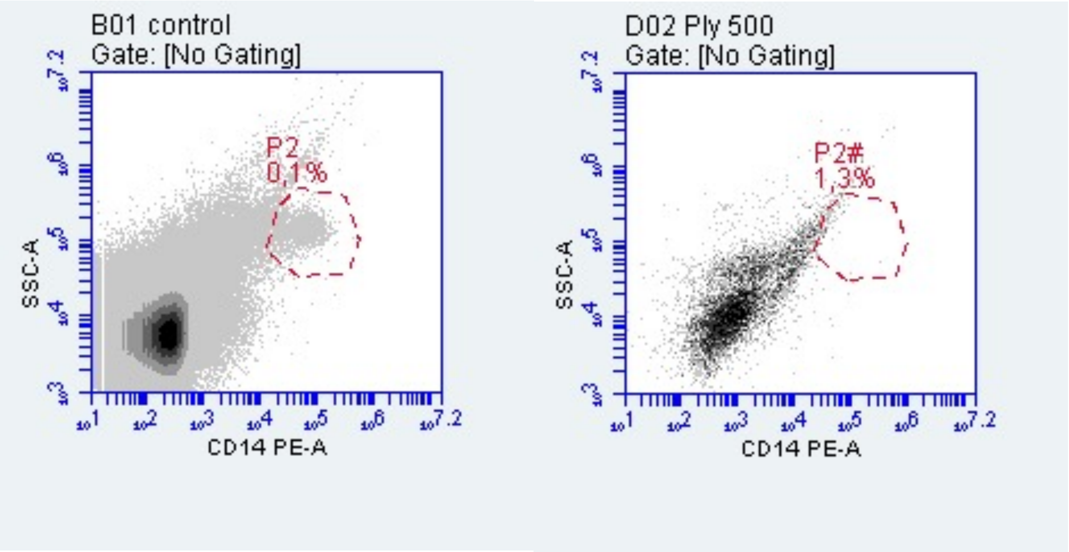

B01 control	Count	Events / µL	%
All	1000000	10000	100
P2	1408	14	100
**D02 Ply 500**	**Count**	**Events / µL**	**% related to control**
All	5502	33	0.5502
P2	72	0	5.113636364

suppl. Figure 3: PBMCs and $CD14^+$ cells after incubation with 0.5 µg/ml pneumolysin (Ply500) or buffer (control) for 20h. Cells were stained with a CD14 antibody and 10^6 cells were analyzed in a FACS Calibur.
