# Supplementary material for: Procoagulant Activity of Blood and Microvesicles Is Disturbed by Pneumococcal Pneumolysin, Which Interacts with Coagulation Factors
Source: J Innate Immun. 2022 Jul 15;15(1):136–52. doi: 10.1159/000525479 (PMC10643893; doi:10.1159/000525479)
Supplement: Supplementary file 4 — Supplementary data [file jin-0015-0136-s04.pdf]

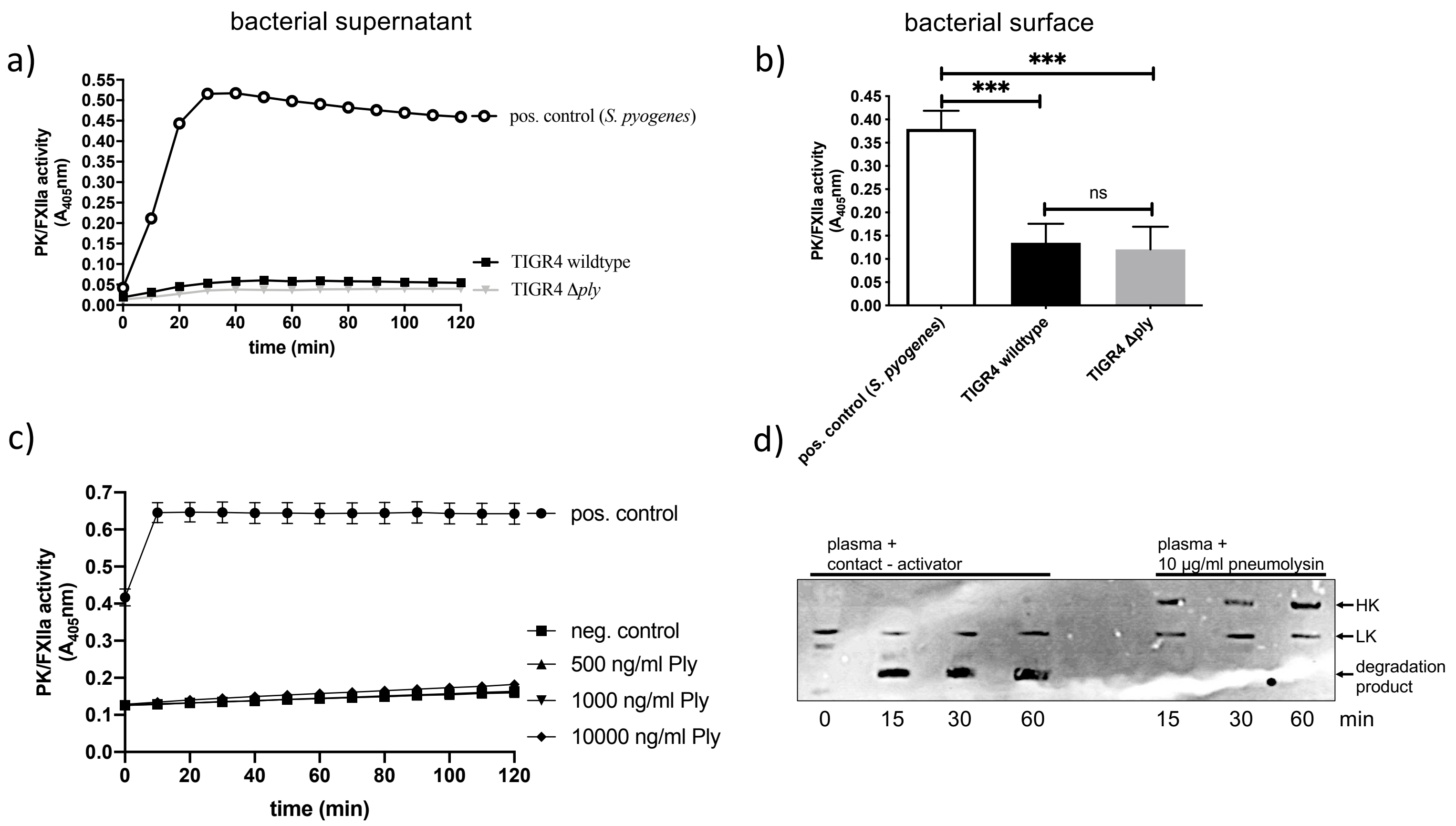

suppl. Figure 4: Contact system activation by pneumococci or purefied pneumolysin

a) PK-activity in plasma after addition of bacterial supernatant from TIGR4 WT and Δ*ply* was measured with the substrate S-2302, that is cleaves by PKa. As positive control the supernatant from *Streptococcus pyogenes* was used. No PK-activity was measured after addition of pneumococcal supernatants.

b) PK-activity detected on the bacterial surface. Bacteria were incubated in plasma, washed and incubated with the substrate S-2302. As positive control *Streptococcus pyogenes* was used. No specific PK-activity was measured with pneumococci. Significance was calculated using one-way Anova, with Dunnetts posttest, ***p < 0.001

c) Pneumolysin was added to plasma with final concentration from 0.5 to 10 µg/ml. The substrate S-2302 was added and the absorbance at 405 nmwas detecetd over 120 min. As positive control (pos. control) a contact system activator (Dapptin) was added. No activation after addition of pneumolysin was measured.

d) Pneumolysin with a final concentration of 10 µg/ml or a contact activator (Dapptin) were incubated in plasma and samples were taken after 0, 15, 30 and 60 min. After SDS-page and Western Blotting HK was stained with an antibody that detects HK at 120 kDa, LK at 60 kDa and a HK-degradation product, visible after contact activation. Addition of the contact activator degraded HK within seconds, and HK-degradation products becomes visible after 15 min of incubation. Addition of pneumolysin did not induce HK-degradation within 60 min.
